# Microbiomes of fish, sediment and seagrass suggest connectivity of coral reef microbial populations

**DOI:** 10.7717/peerj.10026

**Published:** 2020-09-21

**Authors:** Rosa León-Zayas, Molly McCargar, Joshua A. Drew, Jennifer F. Biddle

**Affiliations:** 1 Department of Biology, Willamette University, Salem, OR, USA; 2 Department of Biological Sciences, Fordham University Bronx, NY, NY, USA; 3 Environmental Science and Forestry, State University of New York College of Environmental Science and Forestry, Syracuse, NY, USA; 4 School of Marine Science and Policy, University of Delaware, Lewes, DE, USA

**Keywords:** Microbiome, Coral reef, Fish gut, Sediment

## Abstract

The benthic environments of coral reefs are heavily shaped by physiochemical factors, but also the ecological interactions of the animals and plants in the reef ecosystem. Microbial populations may be shared within the ecosystem of sediments, seagrasses and reef fish. In this study, we hypothesize that coral reef and seagrass environments share members of the microbial community that are rare in some habitats and enriched in others, and that animals may integrate this connectivity. We investigated the potential connectivity between the microbiomes of sediments, seagrass blades and roots (*Syringodium isoetifolium*), and a seagrass-specialist parrotfish (*C. spinidens*) guts in reef areas of Fiji. We contrasted these with sediment samples from the Florida Keys, gut samples from surgeonfish (*A. nigricauda*, *Acanthurinae* sp. unknown, *C. striatus*), and ocean water microbiomes from the Atlantic, Pacific and Indian Oceans to test the robustness of our characterizations of microbiome environments. In general, water, sediment and fish gut samples were all distinct microbiomes. Sediment microbiomes were mostly similar between Fiji and Florida, but also showed some regional similarities. In Fiji, we show connectivity of a shared microbiome between seagrass, fish and sediments. Additionally, we identified an environmental reservoir of a surgeonfish symbiont, *Epulopiscium*. The connection of these ecosystem components suggests that the total microbiome of these environments may vary as their animal inhabitants shift in a changing ocean.

## Introduction

Marine microbes play an important role in the ecology of coral reef systems, including mediating algal:coral interactions (Smith et al., 2006) and providing settlement cues (Ainsworth, Vega Thurber & Gates, 2010). In coral reef-associated systems, such as seagrass beds, there is a complex relationship between the seagrass plants and the microbial community, since seagrasses can release oxygen through their roots and impact local biogeochemistry (Duarte, Holmer & Marbà, 2005). Coral reef fish interact with the surrounding water and feed on seagrass or benthic material, which influences their gut microbiome (Smirga, Sandin & Azam, 2010; Clements et al., 2014). Our understanding of the relationships among the microbial community, the living structural elements (corals, seagrasses etc.) and the associated fish community are limited, yet we know that microbial linkages should permeate classical trophic interactions in reef systems.

Some ecological interactions are known for microbial connectivity in these systems, for example, the algae, *Symbiodiniaceae* spp. that forms the basis of photosynthetic activity for scleractinian corals, is dispersed via the guts of coral-eating fish through their defecation (Castro-Sanguino & Sánchez, 2012). It has also been shown that fish gut microbiomes shift upon fish settlement into a reef system (Parris et al., 2016). Yet little is known about the microbiomes of reef sediments, which are important for nutrition in both fish (Wilson et al., 2003) and invertebrates (Uthicke, 1999). In seagrass and coral reef communities, microbial composition plays a leading role in disease defense and carbon sequestration (Asmus & Asmus, 2000; Küsel et al., 2006; Gantar et al., 2011; Gil-Agudelo et al., 2007), and herbivorous fish grazing is critical to ecosystem health (Heenan & Williams, 2013).

Little is known about how divergent fish gut microbiomes are across species and genera (Clements et al., 2014). Fish, including parrotfish (Scarine members of Labridae; herbivores in this study) and surgeonfish (Acanthuridae; detritovores in this study), are species highly characteristic of coral reef ecosystems (Frydl & Stearn, 1978; Polunin, Harmelin-Vivien & Galzin, 1995). Similar microbial communities have been seen in parrotfish and surgeonfish guts, with major differences being seen based on diet types, across algavores and zooplankton feeders compared to detritivores and omnivores (Clements et al., 2014). Initial studies investigating fish gut microbial diversity examined a variety of feeding strategies, and saw differentiation across diet types, but did not take into account different portions of the gut, which may have distinct microbiomes (Miyake, Ngugi & Stingl, 2015). Notably, surgeonfish also have symbiotic gut microbes, *Epulopiscium*, that have no known environmental reservoir (Flint et al., 2005; Grim, Nemeth & Montgomery, 2013). Examination of the microbiomes of waters, food sources, fish guts and the sedimentary environment will allow us to understand what role fish may play on dispersal and connectivity among microbial communities, including the potential for symbiont dispersal (Grim, Nemeth & Montgomery, 2013).

Here we investigate this ecological interaction over multiple spatial, ecological, and phylogenetic scales. We hypothesize that coral reef and seagrass environments, and the animal hosts that interact in them, have a connected microbiome, where shared members of the microbiome are rare in some habitats within the reef and enriched in others. We predict that interactions within the reef ecosystem will dictate the degree of microbial connectivity. We first examine regional trends, comparing the systems within our study to multiple waters sampled near other coral systems, to show the distinction of these habitats as individual microbiomes. Next, we look at microbial communities at a local biogeographic scale and compare the microbial diversity of inshore reefs in both Fiji and Florida. Lastly, we track the connectivity in microbial similarities among a three-group system in Fiji (seagrass blades and roots, the sediment that seagrass was growing in, and the digestive microbial community of a seagrass eating parrotfish) as well as the digestive microbial community of three species of detritivore surgeonfish collected from the reefs and seagrasses in coastal Fiji. In this system, we see there is an environmental reservoir of the surgeonfish gut symbiont, *Epulopiscium*, which has previously been undetected. In total, we show that these distinct microbiome environments have shared microbial components, which suggests a connected ecosystem and dispersal pathway for the microbes in coral reef environments.

## Materials and Methods

### Sample collection

Sediments from the Florida Keys were collected via diving, with locations either in spur and groove sedimentary structures or near sponges (five samples; Table 1). Samples were taken by hand via push coring using 60 ml cutoff sterile syringes and immediately frozen at −20 °C in the field and −80 °C in the laboratory until analysis. Several locations at two different sites within Fiji were sampled (Table 1): sediments from seagrass and corals near the village of Nagigi on the island of Vanua Levu (two samples), a parrotfish (one fish sampled), *Calotomus spinidens* (Quoy & Gaimard, 1824), from the same seagrass meadows in Nagigi, and surgeonfishes (three fish sampled) (*Ctenochaetus striatus* (Quoy & Gaimard, 1825); *Acanthurus nigricauda*, Duncker & Mohr (1929) and *Acanthurinus* sp. unknown), sediments (four samples) and seagrass (two samples) from a backreef area near the village of Nabukavese on the island of Viti Levu. The Ministry of Education, National Heritage, Culture and Arts (Reference: RA17/13); Fiji Locally Managed Marine Areas (FLMMA) Network, National Trust of Fiji/Conservation International Fiji Office; and the Fiji Immigration Department, Ministry of Defense, National Security and Immigration Office granted approval to access the study site and conduct research. All sampling was conducted with the permission of the community owners of the reefs. All fish were collected under the auspices of the Columbia University Animal Care Board permit to Joshua Drew (ACAAAF6300). The *C. spinidens* is a strictly herbivorous parrotfish, whereas the surgeonfishes collected are detritivores. Fish and seagrass identifications were made visually. Sediments and seagrass were push cored by hand, and fish were dissected immediately and their gut contents stored separately. These samples were frozen in liquid nitrogen and transported to the University of Delaware, where they were stored at −80 °C. Local waters were unable to be sampled during fieldwork.

**Table 1:** Sample codes and descriptions.

Sample type	Habitat	Sample*	Sample key	Water depth (m)	Latitude	Longitude	Collection date	Number of raw sequences***
Backreef Fiji	Sediment core A	top, middle, bottom	SA1-3	1.5	−16.805667°	179.476633°	7/5/13	SA1 113,451; SA2 113,431; SA3 107,680
	Sediment core B	top, middle, bottom	SB1-3	1.5	−16.805667°	179.476633°	7/6/13	SB1 110,011; SB2 121,449; SB3 92,414
	Seagrass sediment core A	top, middle, bottom	SSA1-3	1.5	−16.805667°	179.476633°	7/7/13	SSA1 114,716; SSA2 129,864; SSA3 129,947
	Seagrass sediment core B	top, bottom	SSB1-2	1.5	−16.805667°	179.476633°	7/8/13	SSB1 124,605; SSB2 123,083
	Seagrass from seagrass sediment core A	Blades with more epibionts	GA1	1.5	−16.805667°	179.476633°	7/9/13	123,505
		Blades with fewer epibionts	GA2	1.5	−16.805667°	179.476633°	7/10/13	212,076
		top roots	GA3	1.5	−16.805667°	179.476633°	7/11/13	148,213
		bottom roots	GA4	1.5	−16.805667°	179.476633°	7/12/13	148,360
	Seagrass from seagrass sediment core B	Blades with more epibionts	GB1	1.5	−16.805667°	179.476633°	7/13/13	110,100
		Blades with less epibionts	GB2	1.5	−16.805667°	179.476633°	7/14/13	221,734
		top roots	GB3	1.5	−16.805667°	179.476633°	7/15/13	220,146
		bottom roots	GB4	1.5	−16.805667°	179.476633°	7/16/13	118,076
Forereef Fiji	Sediment core C “deep”	top, bottom	SC1-2	10	−16.811117°	179.476700°	7/17/13	SC1 154,002; SC2 143,924
	Sediment core D “deep”	top, bottom	SD1-2	35	−16.811117°	179.476700°	7/18/13	SD1 120,623; SD2 137,241
Florida Keys	Sediment under *X.muta* sponge	top	R	20.6	24.94999°	80.45377°	6/14/12	114,945
	Sediment under blue vase sponge	top	B	20.6	24.94999°	80.45377°	6/15/12	102,052
	Sediment from floor of spur and groove	top	G	20	24.94999°	80.45377°	6/16/12	113,420
	Sediment at apex head of spur and groove	top	O	18.3	24.94999°	80.45377°	6/17/12	64,883
	Sediment from floor of spur and groove; c = coarser shell area, f = finer grains	top	Yc, Yf	20.6	24.94999°	80.45377°	6/18/12	Yc 128,676
								Yf 105,612
Fish Fiji	Coral backreef	*Ctenochaetus striatus*	S1-4**	>5	−18.222340°	178.26613°	7/5/13	S1 138,000; S2 43,411; S3 74,584; S4 50,021
	Coral backreef	*Acanthurus nigricauda*	C1-2**	>5	−18.222340°	178.26613°	7/5/13	C1 41,664; C2 122,898
	Coral backreef	*Acanthurus sp*.	F1-2**	>5	−18.222340°	178.26613°	7/5/13	F1 115,295; F2 94,403
	Seagrass	*Calotomus spinidens*	P1-4**	1.5	−16.805667°	179.476633°	7/2/13	P1 154,614; P2 152,105; P3 57,777; P4 68,168

**Notes:**

* Top, middle, bottom refer to individual 3 cm sections of sediment or roots; top: 0–3 cm; middle: 3–6 cm; bottom: 6–9 cm.

** For fish C and F (1 = crop and 2 = gut). For fish S and P (1 = crop in S and pharyngeal mill in P, 2 = foregut, 3 = midgut, 4 = hindgut).

*** For statistical analyses, samples were rarefied at a level of 37,000 sequences.

During sectioning, seagrass blades and roots were isolated from the two shallow cores and four categories were created: tops of roots (grass surface to 1 cm), bottoms of roots (>1 cm below grass surface), blades with visual epibionts and blades without visual epibionts (Table 1). Epibionts were important to subsample, as it has been shown that their presence dramatically alters organic matter composition of seagrass beds, and may alter microbial composition as well (Spivak et al., 2009).

Sediments were sectioned into 1 cm slices and labeled based on depth (1 is surface to 1 cm, 2 is 1–2 cm and 3 is 2–3 cm). The outside of the core was discarded and 0.5 g of sediment was added to a DNA extraction tube (see below). Sediments were classified during sampling by visual inspection. In general, Florida samples were fine-grained, whereas Fijian sediments ranged from coquina (SD1-2) to mixed fragmented shells and coarse grains (SC1-2) to finer grains/sand (all others).

For fish samples, the intestinal tract and pharyngeal mill/crop were removed intact, and the intestinal length was measured to subsample into foregut, midgut and hindgut based on length when possible. We desired to separate the gut sections as environmental material would be expected to degrade after intake and digestion along the gut. All samples were taken using sterile methods and placed into sterile Eppendorf tubes prior to DNA extraction.

### DNA extraction and sequencing

DNA extractions of all samples were done according to the instructions in the PowerSoil DNA Isolation Kit from MoBio (Carlsbad, CA, USA). Samples were measured by Nanodrop (ThermoFisher Scientific, Carlsbad, CA, USA). Successful amplification of the bacterial 16S rRNA gene (primers 8F-1492R) was checked on a 1% agarose gel, alongside an extraction blank. Samples were successful and the extraction blank failed as expected. Full amplicon sequencing was performed at the Molecular Research DNA lab (Shallowater, TX, USA) using bacterial primers 27F-519R, with the barcode on the forward primer. A total of 30 cycles of PCR were performed using HotStarTaq Plus Master Mix Kit (Qiagen, Valencia, CA, USA) under the following conditions: 94 °C for 3 min, followed by 28 cycles of 94 °C for 30 s, 53 °C for 40 s and 72 °C for 1 min, and a final elongation step at 72 °C for 5 min. Samples were pooled in equal proportions and purified using calibrated Ampure XP beads. Then the purified PCR product was prepared for sequencing via Illumina TruSeq DNA library preparation protocol and sequenced on the MiSeq platform (Illumina, San Diego, CA, USA). Sequence reads were joined via the Molecular Research pipeline.

### Sequence analysis

The amplicon data was processed with the QIIME software package, where samples were demultiplexed, quality trimmed based on default values (Caporaso et al., 2010). Sequences ranged in number per sample from 41,664 to 226,000 (Table 1), with an average sequence length of 494 nucleotides. Demultiplexed data is available from GenBank accessions: study PRJEB10911; sequence ERS850335. Additional data for ocean water was collected from previous studies that also performed Illumina 16S rRNA bacterial gene sampling: SRR556134, Malaysia (Chan et al., 2013); SRR2015541 IndOce1, SRR2015543 IndOce2, SRR2015540 IndOce3, SRR2015545 IndOce4, SRR2015526 IndOce5 (Jeffries et al., 2015); SRR1693227 MedOce1, SRR1713896 MedOce2, SRR1713895 MedOce3 (Ribes et al., 2015); ERR1103346 AtlaOce1, ERR1103341 AtlaOce2, ERR1103335 AtlaOce3, ERR1103091 AtlaOce3, ERR1103091 AtlaOce4, ERR1103086 AtlaOce5, ERR1103080 AtlaOce6, ERR1103221 AtlaOce7, ERR1103210 AtlaOce9 (Milici et al., 2016). For analysis of samples internal to this study, sequences were clustered into operational taxonomic units (OTUs) by 97% sequence identity using UCLUST (Edgar, 2010). For analyses requiring inclusion of all sequences, sequences were clustered via the closed OTU picking pipeline in QIIME (pick_closed_reference_otus.py) using the default parameters since different primers were used across studies. Representative OTUs were assigned taxonomic classification and aligning to the SILVA database v108 (Quast et al., 2013) using QIIME. The table of representative OTUs was exported into metagenomeSeq software for further analysis (Paulson et al., 2013). Bar graphs of taxonomic abundance at the phylum level were generated from normalized OTU counts. Using sequences seen at least two or more times, or at times sequences seen over 10 times or excluding archaeal reads (see figure legends for details), heat maps and relationships between the different samples were calculated using the Bray–Curtis and Kulczynski dissimilarity indices with second square root transformed data using the vegan R package at three taxonomic levels: phyla, class and OTU level. Archaeal sequences were removed for analyses that only required bacterial sequence comparison. Rarefactions of data from this study were created utilizing the QIIME script with a subsample of 37,000 sequences. Shared (core) microbiome OTUs were calculated based on OTU (97% relatedness) presence in 95% of samples, not including the oceanic water samples, using the compute_core_microbiome.py script in QIIME. Multidimensional scaling plots using a Bray–Curtis statistical metric were generated using the vegan R package.

### Analysis of *Epulopiscium* sequences

Sequence reads identified as *Epulopiscium* in the QIIME taxonomy table were selected. Heatmaps of the abundances of these reads across samples were produced in R. Sequences were compared via BLAST homology to the nt database at NCBI to find relatives. Relative sequences were aligned by the SILVA SINA aligner (Pruesse, Peplies & Glöckner, 2012) and a maximum likelihood tree was created in FastTree v2.1.4 (Price, Dehal & Arkin, 2010). Read sequences were added to the tree via pplacer since they were so short in comparison to relative sequences (Matsen, Kodner & Armbrust, 2010).

## Results

### Microbial diversity

**Figure 1:**
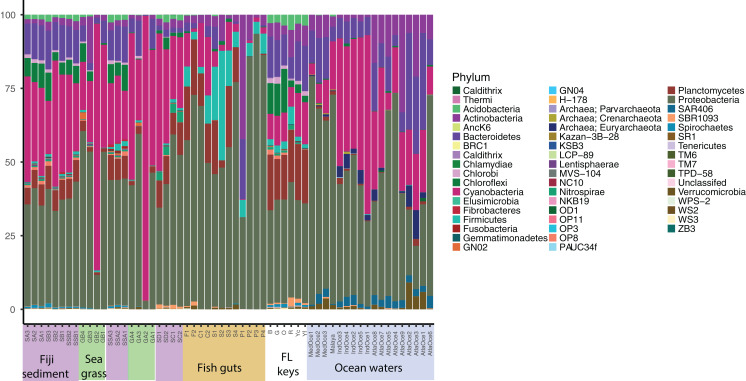
Phylum level diversity from 16S rRNA amplicon sequencing across all samples studied. Percent abundances of each phylum, based on a total of 37,000 sequences per sample, are shown. Sample codes are listed in Table 1.

Relative abundance at the phylum level suggests a few groups are abundant across samples and that similar sample types host similar communities (Fig. 1). Proteobacteria were numerous in all samples except for two seagrass samples, GA2 and GB2, where Cyanobacteria were dominant. It had been visually noted that these two seagrass samples had fewer epibionts (Table 1). Cyanobacteria were present across sediment and ocean samples, and decreased in abundance along the fish guts. Both fish guts and Florida sediments had appreciable numbers of Planctomycetes, which were less abundant in the other sample types. Chloroflexi were found in sediments and ocean waters, but to note, the water Chloroflexi were from the group SAR202, which is abundant in ocean waters (Morris et al., 2004) and the sediment Chloroflexi were from the *Dehalococcoides* genus, which is abundant in sediments (Hug et al., 2013). Archaea were only found appreciable numbers in ocean samples, as the primers used for these studies were broader than the ones used for the other samples.

### Community relationships

**Figure 2:**
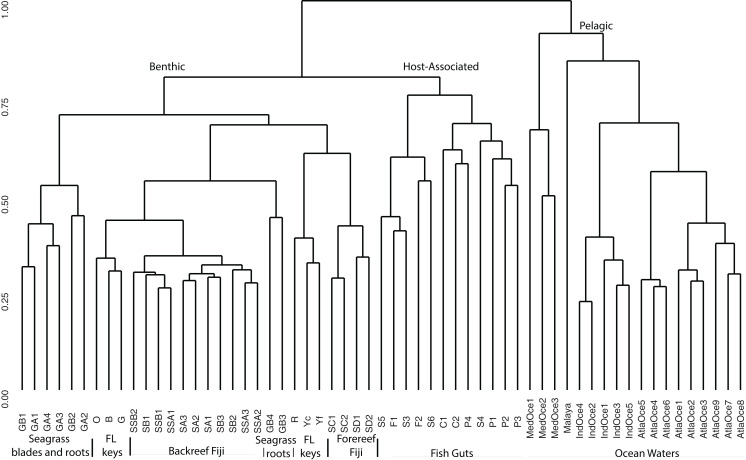
Cluster analysis based on Bray Curtis dissimilarity matrix of all samples analyzed. OTUs were formed at the 97% similarity level using closed picking. Clear separation is seen for benthic, host-associated and pelagic habitats.

For a broad view of community similarity, and to take into account that our samples come from different environments in size and type (e.g., gut vs. water vs. sediment vs. seagrass blades or roots) and had been sequenced with different primers, we used the closed OTU phylogenetic assignments to cluster the samples via the Bray–Curtis dissimilarity index with square-root transformed data (Fig. 2). This showed the oceanic waters clustering by region, separate from the benthic-related samples of Fiji and Florida. Within this benthic cluster, the host-associated fish gut samples clustered together, Florida and Fiji samples were similar yet distinct, and seagrass samples were distinct. The backreef Fijian samples clustered together, separate from the forereef Fijian samples. A portion of the FL sediments clustered with the backreef Fiji samples, and the other portion clustered with the forereef. Two seagrass root samples clustered with backreef samples, while the others were a distinct group within the benthic clade. Since one major group that differed across these samples was Archaea (Fig. 1), we continued to analyze the data under the closed OTU taxonomy, removing the archaeal signatures and only comparing the bacterial taxa for further analyses.

**Figure 3:**
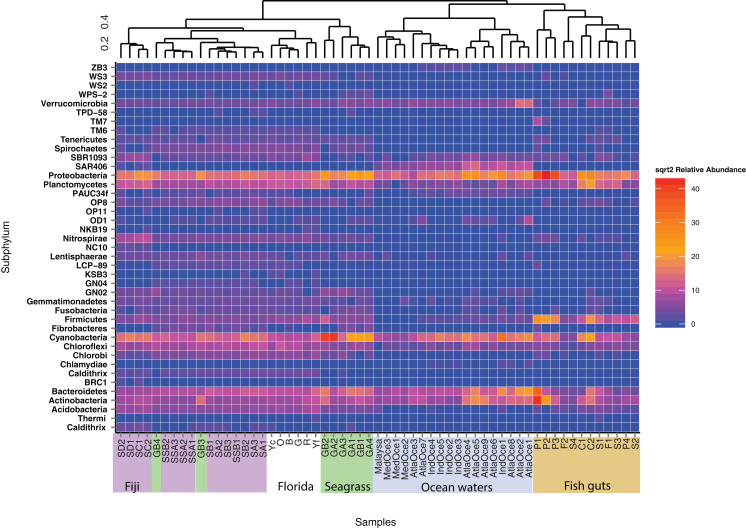
Heatmap of phyla abundance across a cluster matrix based on Kulczynski dissimilarity. Abundances are normalized by second square root transformation, with highly abundant species in red and lowest abundance in blue. Abundances are only considered for OTUs seen over 10 times. All samples are included, archaeal sequences are removed.

To further delve into the details of the benthic (sediment/seagrass):water:fish relationship, we used the Kulczynski dissimilarity index with square-root transformed data to assess statistically significant patterns at the phylum level (Fig. 3). The oceanic waters still group together, but now clade with the fish gut samples. The fish gut samples have noticeably higher levels of Proteobacteria, Firmicutes, Bacteriodetes and Actinobacteria than other environments. Combining samples from different diet preferences and species, the physical location within the gut did not determine the clustering arrangement of the gut samples. Just outside the larger sediment group, the seagrasses, along with top roots, form a distinct cluster (Fig. 3). The sediment samples from Florida showed cohesion, within the larger sediment group, which also houses a distinct top root sample, GB3, along with bottom roots. The Fijian forereef and backreef are still clustered separately. Particularly noticeable in this analysis is that the sediment cluster contains a higher number of different taxa at varying abundances, compared to oceanic and gut samples. We removed the ocean waters from further analysis and used open OTU calling methods to assess the diversity within the remaining dataset.

**Figure 4:**
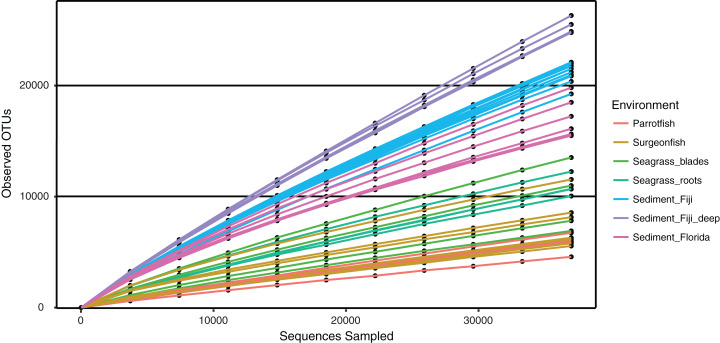
Rarefaction curves of observed OTUs, clustered at 97% similarity, across increasing numbers of subsampled sequences. Shown are deep water Fiji sediments (SD1, SD2, SC1, SC2; purple), shallow water Fiji sediments (turquoise), Florida sediments (pink), seagrass blades (green), seagrass roots (blue green), Surgeonfish (beige) and the herbivorous Parrotfish *C. spinidens* (orange).

Microbial alpha diversity (i.e., rarefaction) was calculated by randomly sampling a subset of 37,000 sequences from all remaining samples (Table 1). The rarefaction curves suggest that all environments were not sampled to exhaustion, as curve saturation was not reached (Fig. 4). However, a clear pattern of species richness shows that sediment samples were the most diverse when compared to seagrass blade, root and fish samples (Fig. 4). Within this study, Fijian sediments (sediment_Fiji and sediment_Fiji_deep) were more diverse when compared to Floridian sediments (sediment_Florida). Within the Fijian sediments, the sediments collected at a deeper site in the forereef (sediment_Fiji_deep) were more diverse than shallow samples (sediment_Fiji) in the backreef. Seagrass roots and blades have a mid-level of total diversity, between sediments and fish guts. In general, animal-associated habitats had the least diversity; with the herbivorous *C. spinidens* parrotfish gut having the lowest overall diversity.

### Connectivity of environments

**Figure 5:**
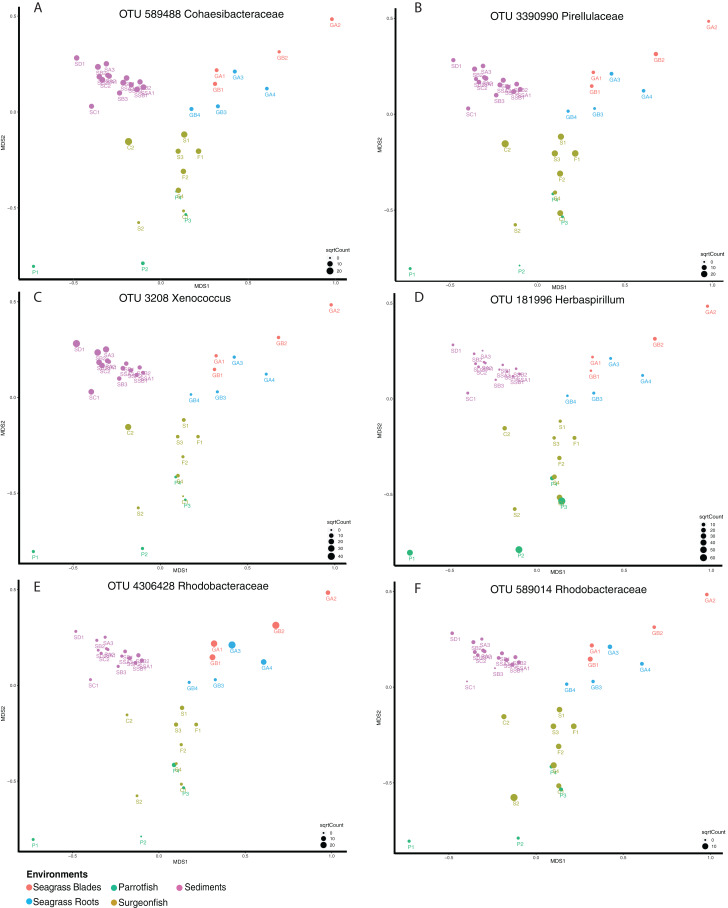
Shared microbiome member abundance across samples. Individual OTU bubble plots are shown by multidimensional scaling based on Bray–Curtis dissimilarity of the shared microbiome. Each panel has its own index for bubble size, relating to abundance. Samples are coded by color: seagrass blades (red), seagrass roots (turquoise), Surgeonfish (brown), Parrotfish (C. spinidens) (green), deep Fiji sediment (purple), shallow Fiji sediment (pink). (A) OTU 589488 Cohaesibacteraceae, (B) OTU 3390990 Pirellulaceae, (C) OTU 3208 Xenococcus, (D) OTU 181996 Herbaspirillum, (E) OTU 4306428 Rhodobacteraceae, (F) OTU 589014 Rhodobacteraceae. Actual abundances are shown in Table S1, highlighted examples are bold in that table. Florida samples are not shown since they were not physically connected to Fiji samples and as such were not considered in this analysis.

Shared microbiome analyses were generated for the Fijian samples in order to better understand the connectivity between the samples based on microbial distribution. While this utilized the “core microbiome” analysis method, we opt to term this a “shared microbiome” considering that multiple habitats are being included in the analysis, whereas a “core microbiome” is typically determined for one type of habitat (Shade & Handelsman, 2011). For this analysis, we used open OTU calling, with 97% similar sequences being called an OTU. This analysis was conducted at a 95% shared threshold, meaning that an OTU was considered to be part of the shared microbiome if it was present in 95% of all samples. From the analysis we found that 34 OTUs are distributed among the shared microbiome (Table S1), including Proteobacteria (17), Cyanobacteria (including chloroplasts) (9), Actinobacteria (2), Bacteroidetes (1) and Planctomycetes (1). While the OTUs within the core microbiome are shared by 95% of the samples, the relative abundance of each OTU per sample is variable (Fig. 5). We highlight six OTUs that have differential distribution in the habitats. OTUs from *Cohaesibacteracaea* and *Pirulleaceae* are most abundant in surgeonfish crops and sediments (Fig. 5AB). *Xenococcus* is found in sediments and a surgeonfish gut (Fig. 5C). *Herbaspirillum* is most concentrated in parrotfish gut and seagrass blades (Fig. 5D). A *Rhodobacteraceae* OTU is most abundant in seagrass (Fig. 5E) and a different *Rhodobacteraceae* OTU is most abundant in surgeonfish (Fig. 5F).

**Figure 6:**
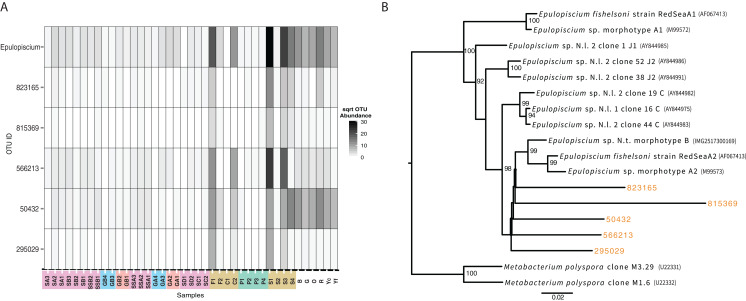
Analysis of the surgeonfish symbiont, *Epulopiscium*, seen across all samples. (A) Heatmap showing abundance across samples by total and per OTU. Color codes follow from Fig. 5. (B) Maximum likelihood phylogenetic tree of 16S rRNA genes from known taxa and new OTUs seen in this study, which were added with pplacer. Names of denovo OTUs are the same as shown in (A). Accession numbers are in parentheses.

While not detected as a member of the shared microbiome, another taxon that was highly prevalent in the mid and hindguts of the surgeonfish, *Acanthurus* sp., *Acanthurus nigricauda* and *Ctenochaetus striatus* was *Epulopiscium* (Fig. 6). This taxon known as a symbiont of surgeonfish (Fishelson, Montgomery & Myrberg, 1985) and is one of the largest bacteria ever seen. While it is most abundant in the surgeonfish samples, similar sequences are seen in multiple benthic environments, particularly in the Florida samples. The overall signature of *Epulopiscium* is most abundant in samples F1, C2, S1, S3 and S4, which are surgeonfish crops (F1, S1), and guts (C2, S3, S4) (Fig. 6; Table 1 shows sample codes). Very few sequences are seen in the parrotfish gut samples (P1-4) (Fig. 6). Five different distinct OTUs are seen, with differing concentrations across the gut regions and fish species, and one of the OTUs, 50432, showing widespread coverage in the Florida sediments in addition to the Fijian fish, suggesting this is a ubiquitous lineage.

## Discussion

Our study examined the microbiome of multiple environments that should interact on a coral reef: the sediments, fish, seagrass and oceanic waters. While our study is limited by a lack of replication, uneven sampling efforts and water samples that were not taken in-situ, we are able to see broad trends in microbiomes and explore details of microbial connectivity across samples. We investigated sediments from both Fiji and Florida reef systems. In general, these sediments are quite similar although local similarity is expected (Ruff et al., 2015; Hanson et al., 2012). The sediment samples consistently grouped together, separate from oceanic waters. Surprisingly, we noted a trend of high diversity within benthic Fiji samples, which mirrors large-scale patterns in macroecology, as the Indo-Pacific harbors a higher diversity of fish, corals, and mangroves (Roberts et al., 2002). Whether microbial trends are driven by the same factors as macroorganisms is unknown, as microorganisms are classically expected to diversify based on local chemical factors (Hanson et al., 2012). We, unfortunately, were unable to collect chemical data on these environments to compare during our study, as time and resources were limited. The driving forces behind the consistently high diversity in Fiji, compared to the reef environment in Florida, are intriguing and should be investigated and compared with other reef systems in future studies.

We observe a general trend of decreased microbial diversity in association with seagrasses (blades and roots) and fish. This agrees with trends seen in more comprehensive studies, that host-associated habitats have the least microbial diversity (Schloss et al., 2016). The strictly herbivorous parrotfish, *C. spinidens*, shows the lowest diversity.

The sediment communities are generally similar between Fiji and Florida, despite different biogeographic provinces and depths, whereas the fish showed distinct populations, both within the fish and across fish species. Within the parrotfish, populations shift along the gut, sometimes showing drastic shifts from the pharyngeal mill through hindgut (samples P1–P4; Fig. 3). There are high numbers of Planctomycetes, Verrucomicrobia and differing abundances of Proteobacteria and other less dominant groups. Overall, the Firmicutes are the most distinct group within the fish cluster, and are present in varying amounts in all samples, except for being distinctly low in the hindgut of *C. striatus*. The contributions of microbial populations to fermentation in herbivorous fish has been contested (Clements et al., 2014), yet the drastically different communities in these regions suggest microbes are actively responding to gut position. However, when we consider the overall relatedness of the samples, we see that in general, despite differences in species and gut location, the fish samples are considered similar to each other, compared to ocean waters, seagrasses, sediments and deep sediments.

While the fish communities are distinct from the environmental samples, they do share common organisms. We examined the shared microbiome across the Fijian samples and see that specific organisms can be tracked between the external environment and the fish. OTUs from the Rhodobacteraceae and Pirellulaceae are abundant in the fish guts and in low abundance in the environment. An OTU from Herbaspirillum was most abundant in seagrass and parrotfish guts, linking the consumer and food source. The drop in abundance in mid and hindguts shows that the fish are likely digesting these organisms, but some signatures make it through. This confirms our hypothesis that a coral reef and seagrass environment contains microbes that are shared across habitats, including their animal hosts, and increase in number in only some habitats. Whether or not these signatures are from live organisms is unknown since this analysis was only performed on DNA and could potentially detect extracellular DNA or dead cells.

Alongside these shared populations, we also noted that the genus *Epulopiscium* was seen across the samples in varying abundances. While it was most abundant in only the surgeonfish guts, where it is widely known as a symbiont (Clements, Sutton & Choat, 1989), it was also seen in many environmental samples, particularly the Florida Keys sediments. Notably, the detection of *Epulopiscium* is in low abundance in the parrotfish sample, and different lineages of *Epulopiscium* dominate each of the surgeonfish species sampled, suggesting these lineages are, as expected, symbiotic in the sampled surgeonfish as the observed pattern suggests species–specific host adaptation. The same exact lineage is seen across multiple environmental samples, and is most abundant in the Florida Keys samples that are most likely to be exposed to fish, near sponges or at the head of spur and groove sediment, which are most likely to be exposed to fish.

The lineages of *Epulopiscium* we found also branch away from known sequences of *Epulopiscium*, suggesting more diversity of this group is present in the environment. The mechanism of transmission of this symbiont has been debated, particularly since it unknown to survive outside of the host system (Flint et al., 2005). An environmental repository of *Epulopiscium* has not yet been defined, although survival outside of the fish could take place via a sporulated form, which would be detectable in our analysis (Flint et al., 2005; Grim, Nemeth & Montgomery, 2013). This study shows that cells of *Epulopiscium* reside in sedimentary environments. This, however, should be taken with the caveat that no activity is defined by this measurement, and the ability for the symbionts to be taken up from the sedimentary environment cannot yet be established. However, it has been shown previously that these symbionts are cleared when fish are starved (Fishelson, Montgomery & Myrberg, 1985; Montgomery & Pollak, 1988), suggesting that active recharging of their symbiont population could be occurring through this sedimentary reservoir. surgeonfish have previously been shown to harbor gut communities that reflect their host dietary preferences and taxonomy (Miyake, Ngugi & Stingl, 2015). However, past studies did not section gut samples or explore the benthic environment for relatedness. Some of the materials from gut communities, such as the symbionts that proliferate in surgeonfish guts, are seen in the benthic community.

## Conclusions

We investigated the microbiomes of oceanic waters near coral reefs, coral reef sediments in Fiji and Florida, and coral reef fish in Fiji. We see that these are distinct microbiomes, however, there is connectivity between physically circulating material, mediated by fish. These highly diverse benthic environments are created by unknown factors, particularly in Fiji. It is likely that chemical factors are likely a large determinant of total populations. However, the benthic environments in these areas are intimately linked with the animal populations of the reefs. With the high potential for change in the animal ecosystem of the fish, these communities should continue to be cataloged. Understanding connectivity among reefs, across multiple geographic and trophic scales remains one of the major challenges in tropical coastal ecology. This work suggests that microbial diversity may mirror larger macroecological processes and that connectivity exists between seagrass, sediments and fish in an intimately connected ecosystem. This small, initial study provides interesting hypotheses to pursue in future studies.

## Supplemental Information

10.7717/peerj.10026/supp-1 Supplemental Information 1 Shared OTUs across samples. Bolded entries are the samples highlighted in Fig. 5.

## Additional Information and Declarations

### Field Study Permissions

The following information was supplied relating to field study approvals (i.e., approving body and any reference numbers): The Ministry of Education, National Heritage, Culture and Arts (Reference: RA17/13); Fiji Locally Managed Marine Areas (FLMMA) Network, National Trust of Fiji/Conservation International Fiji Office; and the Fiji Immigration Department, Ministry of Defense, National Security & Immigration Office granted approval to access the study site and conduct research. All sampling was conducted with the permission of the community owners of the reefs.

### Data Availability

The following information was supplied regarding data availability: DNA sequences are available at NCBI BioProject: PRJEB10911; SRA: ERS850335.
